# Optimizing Bothropstoxin-I-Derived Peptides: Exploring the Antibacterial Potential of p-BthW

**DOI:** 10.1021/acsomega.4c01303

**Published:** 2024-05-22

**Authors:** Gabriela Marinho Righetto, Norival Alves Santos-Filho, Letícia Oliveira Catarin Nunes, Camille André, Julia Medeiros Souza, Adriano Defini Andricopulo, Paulo José Martins Bispo, Eduardo Maffud Cilli, Ilana Lopes Baratella da Cunha Camargo

**Affiliations:** † Laboratory of Molecular Epidemiology and Microbiology, Department of Physics and Interdisciplinary Science, University of Sao Paulo, 13563-120 São Carlos, Brazil; ‡ Department of Biochemistry and Organic Chemistry, Institute of Chemistry, São Paulo State University, 14800-060 Araraquara, Brazil; § Infectious Disease Institute, Department of Ophthalmology, Massachusetts Eye and Ear, Harvard Medical School, Boston, Massachusetts 02115, United States; ∥ Laboratory of Medicinal and Computational Chemistry, Department of Physics and Interdisciplinary Science, University of Sao Paulo, 13563-120 São Carlos, Brazil

## Abstract

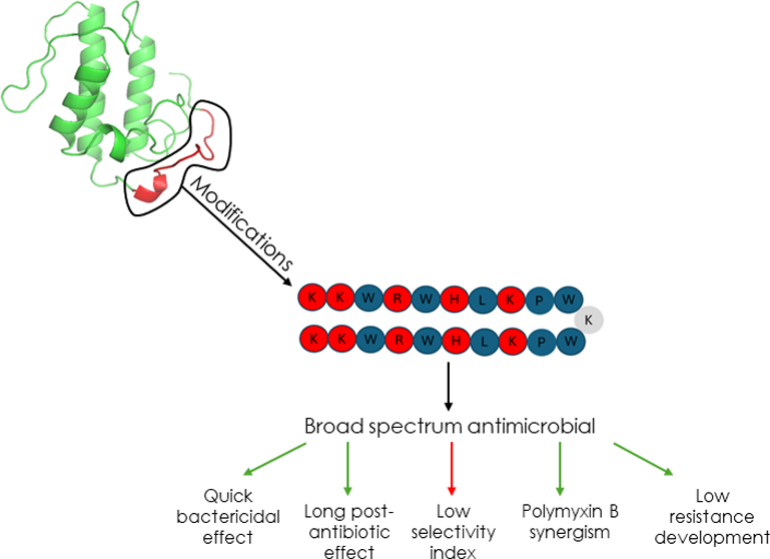

Antimicrobial peptides are an emerging class of antibiotics that present a series of advantageous characteristics such as wide structural variety, broad spectrum of activity, and low propensity to select for resistance. They are found in all classes of life as defense molecules. A group of peptides derived from the protein Bothropstoxin-I has been previously studied as an alternative treatment against multi-drug-resistant bacteria. The peptide p-BthTX-I (sequence: KKYRYHLKPFCKK) and its homodimer, linked by disulfide oxidation through the residues of Cys11 and the serum degradation product [sequence: (KKYRYHLKPFC)_2_], were evaluated and showed similar antimicrobial activity. In this study, we synthesized an analogue of p-BthTX-I that uses the strategy of Fmoc-Lys(Fmoc)-OH in the C-terminal region for dimerization and tryptophan for all aromatic amino acids to provide better membrane interactions. This analogue, named p-BthW, displayed potent antibacterial activity at lower concentrations and maintained the same hemolytic levels as the original molecule. Our assessment revealed that p-BthW has a quick in vitro bactericidal action and prolonged post-antibiotic effect, comparable to the action of polymyxin B. The mode of action of p-BthW seems to rely not only on membrane depolarization but also on necrosis-like effects, especially in Gram-negative bacteria. Overall, the remarkable results regarding the propensity to develop resistance reaffirmed the great potential of the developed molecule.

## Introduction

The CDC recommends four main actions to combat antimicrobial resistance: prevention, tracking, improving antibiotic prescriptions, and developing new tests and drugs.^1^ Although more recent data indicate that antimicrobial resistance will be the leading cause of death in less than 30 years, the development of new drugs has been neglected by the pharmaceutical industry since the 20th century, and no new class of antibiotics has been registered in the market since then.^2,3^ Although natural biodiversity can be considered an almost infinite source of new molecules, only a limited number of natural antimicrobial molecules have been developed into treatments and transitioned to clinical application.^4^

In the search for alternative treatments, research on antimicrobial peptides (AMP) has gained ground in recent years. Among numerous properties, these antimicrobials stand out for their structural diversity, broad spectrum of action, low development of resistance, and synergistic activities.^5^ An AMP of biological importance is p-BthTX-I, which is obtained from the C-terminal portion of the Bothropstoxin-I protein (BthTX-I). Bothropstoxin-I is a 13 700 Da protein isolated from the venom of *Bothrops jararacussu*, a snake native to South America and popularly known as Jararacuçu.^6^ Despite not showing catalytic activity, this protein has structural and molecular similarities to phospholipases A_2_ (PLA_2_).^7,8^ These PLA_2_ are involved in phospholipid metabolism, cell proliferation, and muscle contractions. Studies suggest that some of these PLA_2_-like proteins found in the venom of snakes can prevent pathogenic action in the venom excretory gland, in addition to avoiding the prey of such animals from being consumed by the degradation of other microorganisms—a starting point that led to obtaining p-BthTX-I.^9,10^

A peptide derived from the C-terminal portion of Bothropstoxin-I, sequence KKYRYHLKPFCKK, proved to be an exciting candidate for application as an antimicrobial. Peptide dimerization was used as a rational design strategy to increase activity and selectivity. Initially, the dimer showed antimicrobial activity against *S. aureus* and *E. coli* but not against fungi such as *C. albicans*, with low or no hemolytic activity and toxicity against epithelial cells, macrophages, and erythrocytes at the concentrations tested. When tested in membrane models, the dimer stood out for not causing lysis or even pore formation, unlike some AMPs.^7,8,11^

**Figure 1:**
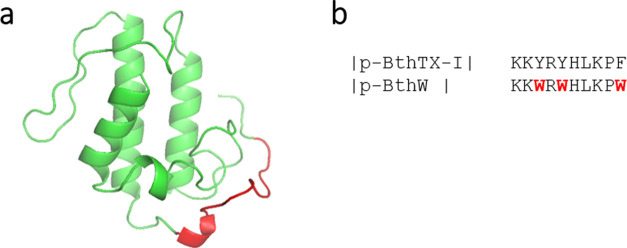
(a) Tertiary structure representation of the PLA_2_ homologue Bothropstoxin-I (PDB ID 3I3H) with details of the C-terminal region of p-BthTX-I between the amino acids corresponding to residues 115–129 in red. Figure was generated by the PyMOL (PyMOL Molecular Graphics System, Version 1.7.4 Schrödinger, LLC.) (b) Alignment between the monomers of previously studied peptide (p-BthTX-I)_2_K and the new modified peptide p-BthW, with amino acid modifications highlighted in red.

A stability study in serum showed the loss of the two lysins in the C-terminal region, probably caused by the activity of carboxypeptidase B. This blood enzyme degrades regions of the peptide that are rich in lysine and arginine. In addition, structural studies have shown the importance of aromatic amino acids in the antimicrobial activity of the peptide, as well as the advantage of using lysine in the C-terminal portion for dimerization. Santos-Filho et al.^12^ synthesized and analyzed a dimeric peptide named des-Cys^11^,Lys^12^,Lys13-(p-BthTX-I)_2_K (or (p-BthTX-I)_2_K) [sequence: (KKYRYHLKPF)_2_K]. This molecule was synthesized using Fmoc-Lys(Fmoc)-OH in the C-terminal region. This strategy was based on the fact that after coupling and deprotection of the first amino acid in the resin, the peptide simultaneously elongates in the two peptide chains. This protocol avoids additional steps to obtain a dimeric peptide, such as Cys residue oxidation, which decreases the time required and the synthesis costs.^12^ Then, in this work, we synthesized and tested the peptide [Trp^3,5,10^] des-Cys^11^, Lys^12^, Lys^13^-(pBthTX-I)_2_K [sequence: (KKWRWHLKPW)_2_K], here named as p-BthW, which is an analogue of the (p-BthTX-I)_2_K, the difference is the replacement of tyrosine residues in positions 3 and 5 and phenylalanine in position 10 by tryptophan. An image providing the location of p-BthTX-I in the protein Bothropstoxin-I is provided in Figure 1, as well as the alignment between the previously studied peptide and the new analogue p-BthW. This amino acid is crucial for proteins and peptides to interact with membranes.^13,14^ This simple exchange vastly increased the activity against Gram-negative bacteria, highlighting the importance of the structure–activity relationship.

## Results and Discussion

### MIC and MBC

The suggested alteration for this peptide was based on the relationship between tryptophan and antimicrobial activity. This amino acid plays a crucial role in proteins and peptides interacting with membranes.^13,14^ In addition, several studies show a relationship between this amino acid and increased antimicrobial activity without increasing hemolytic activity.^14−17^

The initial assessment of the newly synthesized peptide was its comparison with its previously evaluated analogue for antibacterial activity and cytotoxicity. This was performed by determining the MIC for eight well-established ATCC strains and testing their hemolytic activity, as shown in Table 1.

**Table 1:** Antimicrobial Effect of (p-BthX-I)_2_K and p-BthW

peptide	(p-BthTX-I)_2_K^11^		p-BthW	
sequence	(KKYRYHLKPF)_2_K		(KKWRWHLKPW)_2_K	
MW (g/mol)	2869		3040	
HC_50_^a^ (μg/mL)	>512		>512	
minimal inhibitory concentration				
	μg/mL	μM	μg/mL	μM
*S. epidermidis* ATCC 35984	16	5.6	8	2.6
*S. aureus* ATCC 25923	128	44.6	16	5.3
*E. faecalis* ATCC 29212	128	44.6	32	10.5
*E. faecium* ATCC 700221	32	11.1	8	2.6
*K. pneumoniae* ATCC 700603	256	89.2	32	10.5
*E. coli* ATCC 25922	64	22.3	32	10.5
*A. baumannii* ATCC 19606	256	89.2	32	10.5
*P. aeruginosa* ATCC 27583	>512	>178.4	64	21.1

a HC_50_, concentration of 50% hemolysis rate.

The dimerization of the p-BthTX-I peptide at the C-terminal portion has already proven to be an efficient strategy to enhance the antimicrobial activity of this molecule.^12^ In this study, the use of tryptophan in substitution of phenylalanine as an aromatic amino acid increased the activity of the peptide in Gram-negative bacteria. Tryptophan is crucial for proteins and peptides to interact with membranes. Therefore, because one of the main protective mechanisms of Gram-negative bacteria is the outer membrane that prevents the permeability of a series of molecules, the hypothesis is that tryptophan facilitates the interaction and permeability of p-BthW through such a barrier.^18^ The proposed new peptide achieved MICs at least 75% lower for Gram-negative bacteria than the original peptide without losing activity for Gram-positive bacteria. An extensive characterization of the antimicrobial action of the peptide confirmed its broad spectrum of activity against an extensive list of Gram-positive and Gram-negative bacterial strains tested, including those with multiple antibiotic resistance mechanisms (MIC and MBC values are shown in Tables S1 and S2). This modification was also favorable because it maintained the low hemolytic activity of the original molecule.

These results prompted us to further investigate the antimicrobial activity and potential mechanisms of action of this molecule. To do so, two bacterial species were selected. *Staphylococcus aureus* and *Acinetobacter baumannii* were selected as model organisms for Gram-positive and Gram-negative species, representing clinically relevant pathogens. In addition to the ATCC strains, clinical isolates *S. aureus* SA43 (a methicillin-resistant strain^19,20^) and *A. baumannii* ACI50 (a carbapenem-resistant strain^21^) were used as their resistance profiles are on top of the list from WHO for research and development of new antibiotics.^22^ Both strains are well characterized and have their genome available.^19,21^

### Time Kill

**Figure 2:**
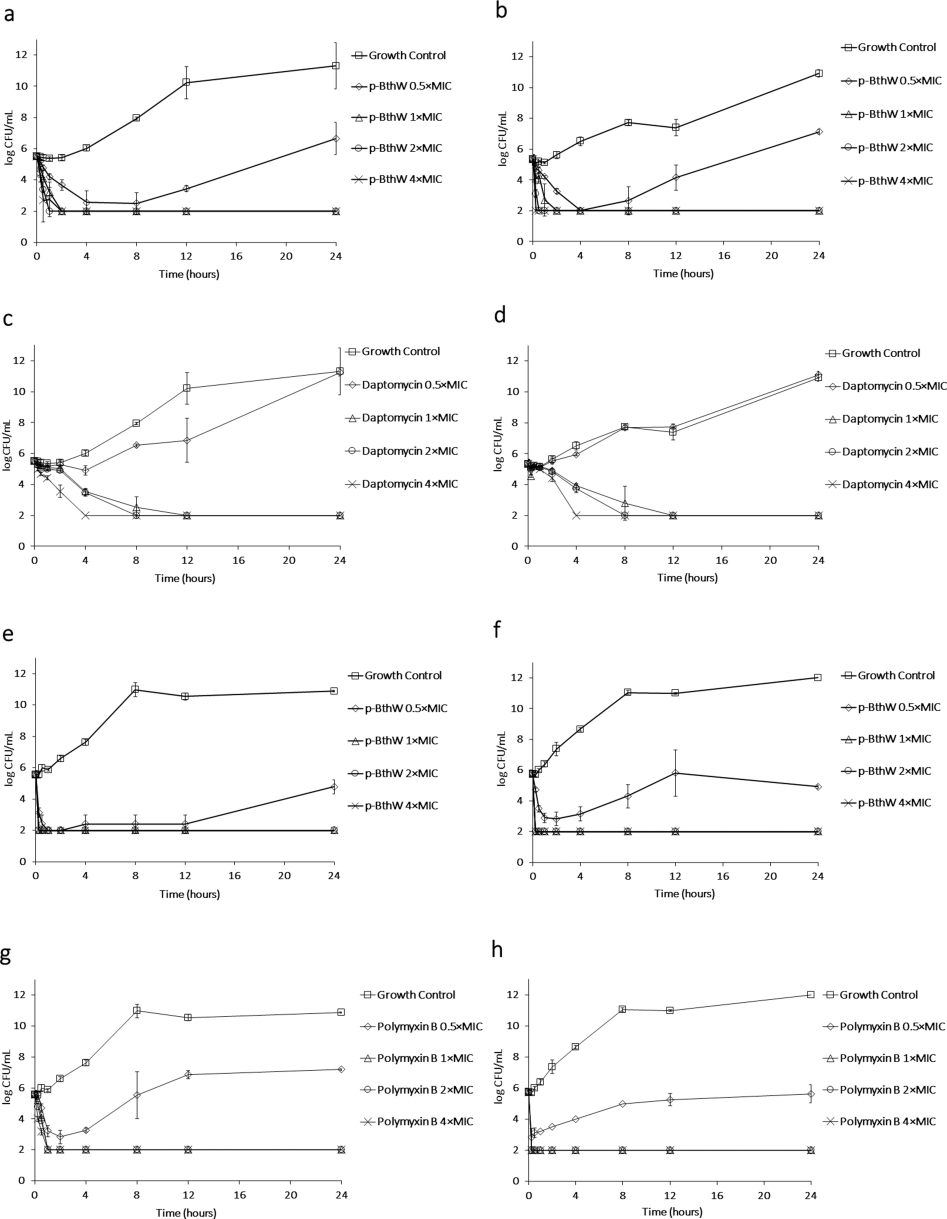
Time-kill of p-BthW against (a) *S. aureus* ATCC 25923 (MIC = 16 μg/mL) and (b) *S. aureus* SA43 (MIC = 16 μg/mL), (e) *A. baumannii* ATCC 19606 (MIC = 32 μg/mL), and (f) *A. baumannii* ACI50 (MIC = 32 μg/mL); daptomycin against (c) *S. aureus* ATCC 25923 (MIC = 1 μg/mL) and (d) *S. aureus* SA43 (MIC = 0.5 μg/mL); and polyxymin B against (g) *A. baumannii* ATCC 19606 (MIC = 0.5 μg/mL) and (h) *A. baumannii* ACI50 (MIC = 128 μg/mL).

A study of death kinetics was carried out to monitor the effect of different concentrations of the p-BthW peptide over time. The results are displayed in Figure 2.

For both *S. aureus* strains, rapid bactericidal activity was achieved by the p-BthW peptide. Within 2 h, it caused a 4-log reduction in the bacterial population, even at the lowest concentrations. When compared to the MIC concentration, the bactericidal activity of p-BthW was faster than that of the commercial antibiotic daptomycin, but it is important to highlight that daptomycin MIC is 16 times lower than p-BthW, which could explain the difference in killing kinetics. Furthermore, even at sub-inhibitory concentrations of p-BthW, there was a large reduction in the microbial population.

As observed for Gram-positive bacterial strains, rapid killing was observed against *A. baumannii*, even at the lowest concentrations. The sub-inhibitory concentration was also able to keep the bacterial population reduced even after 24 h.

Previously studied analogues, such as p-BthTX-I and (p-BthTX-I)_2_, presented similar killing patterns, but cells regained growth after 12 h.^7^ This shows that modifications introduced in the new p-BthW peptide enhanced its antimicrobial activity, ensuring complete microbial killing at drug concentration at or above the MIC.

### Post-Antibiotic Effect

The determination of the post-antibiotic effect (Table 2) was carried out to verify how long the influence of the peptide activity was maintained on the bacterial inoculum. To avoid complete bacterial death in the assay, the time-kill results were crucial once it allowed the selection of a proper exposure time.

**Table 2:** Post-Antibiotic Effect of p-BthW^a^

	post-antibiotic effect of the treatments (h ± s.d.)					
	0.5× MIC			1× MIC		
bacterial strains	p-BthW	daptomycin	polymyxin B	p-BthW	daptomycin	polymyxin B
*S. aureus* ATCC 25923	2 ± 1	0.5 ± 0.5	N.A.	9.0 ± 0.5	1.5 ± 0.5	N.A.
*S. aureus* SA43^19^	5 ± 1	N.O.	N.A.	8 ± 2	3 ± 1	N.A.
*A. baumannii* ATCC 19606	5.5 ± 0.5	N.A.	4.0 ± 0.5	7.0 ± 0.5	N.A.	5.0 ± 0.5
*A. baumannii* ACI50^21^	2 ± 2	N.A.	3.0 ± 0.5	4 ± 1	N.A.	3.0 ± 0.5

a s.d.: standard deviation. N.O.: not observed. N.A.: not applicable.

p-BthW efficiently prevented bacterial regrowth after exposure. In Gram-negative bacteria, the PAE observed for p-BthW is very similar to that obtained for polymyxin B. The results from the time-kill and post-antibiotic effect suggest that the p-BthW peptide can be classified as concentration-dependent: they are more effective if they reach a high concentration, but the time for which this concentration is maintained is less important. Once the intervals of antibiotics in treatment can be essential for its success, the prolonged inhibition associated with rapid cell death may ensure a lower drug administration frequency. This can be an advantage, even more so considering that it has long been recognized in the clinic that patients can take their prescriptions at less frequent intervals than prescribed or discontinue them before completion.^23^

### Cytotoxicity

3-(4,5-Dimethylthiazol-2-yl)-5-(3-carboxymethoxyphenyl)-2-(4-sulfophenyl)-2H-tetrazolium (MTS) cytotoxicity assay was performed as a toxicity measure to investigate whether this peptide could act in eukaryotic cells, complementing the hemolysis study. The selectivity index helps to understand the concentration at which the compound exerts a deleterious effect on human cells compared with the compound minimal inhibitory concentration against the bacteria. The selectivity index is the ratio between the CC_50_ and the MIC.^24^ The SIs for all bacterial isolates used against p-BthW are presented in Tables S1 and S2. For an easier and broader representation of the data, we also used the MIC_50_—the minimum concentration required to inhibit 50% of bacterial strains tested within a given group. MIC_50_ is one of the ways to represent the intrinsic activity of each antimicrobial.^25^ Ideally, to be considered a biologically effective antibiotic, an SI ≥ 10 is expected.^24,26^ The CC_50_ (concentration that reduced cell viability by 50%) of the THP-1 and HFF-1 cell lines and the selectivity index for p-BthW are shown in Table 3.

**Table 3:** CC_50_ and Selectivity Index (SI) for p-BthW

		THP-1		HFF-1	
	MIC_50_ (μg/mL)	CC_50_ (μg/mL)	SI	CC_50_ (μg/mL)	SI
Gram-positives	16	61 ± 1	3	258 ± 4	16
Gram-negatives	32		2		8

When evaluating the bacterial species used, *E. faecalis* had the worst selectivity indexes among the Gram-positive bacteria, while *K. pneumoniae* and *P. aeruginosa* presented the worst selectivity among the Gram-negatives. Comparing all selectivity indices that were evaluated, the fibroblasts presented the most prospective, indicating that topical treatment use might be possible. Even though SI = 8 is not ideal, additional studies could give a better insight into the viability of this application. Alternatively, p-BthW can be combined with other commercial antibiotics to rescue the use of currently less-used drugs.

### Synergism

Determining the potential synergistic activity of the new peptide with currently available antibiotics could support further investigations on the use of combined therapies employing the minimum needed amount of peptide necessary to kill bacteria, which would in turn optimize its SI scores. No synergism or antagonism was observed with other antibiotics against Gram-positive bacteria (*S. aureus* ATCC 25923 used for the assay, see Table S3). For *A. baumannii*, on the other hand, a lower FIC was observed (Table 4).

**Table 4:** Synergism for p-BthW in *A. baumannii* ATCC 19606 (MIC = 32 μg/mL)^a^

		combination (μg/mL)		
antibiotics	antibiotics MIC (μg/mL)	MIC_antibiotic_	MIC_p-BthW_	FIC index
ciprofloxacin	1	0.5	4	0.6
tobramycin	4	1	8	0.5
polymyxin B	1	0.12	2	0.2
vancomycin	>64	>64	32	N.D.
ampicillin	>64	>64	32	N.D.

a N.D. Not determined.

The new p-BthW peptide presented a lower MIC than the previously tested analogues against *A. baumannii*, and its antimicrobial activity was further enhanced in the presence of polymyxin B. Lower FIC for the Gram-negative organism represents an advantage since these organisms are more tenacious, and their infections are considerably more difficult to treat. p-BthW did not show any antagonism among the antibiotics tested, which means that this molecule does not act competitively for the same targets as the tested antibiotics. Resistance to polymyxins has been a growing concern in the clinic and frequently appears in the priority organisms on the WHO list for research and development of new drugs.^22^ Therefore, the synergism of the peptide with this antibiotic is of great interest as it is an opportunity to potentially rescue the activity of polymyxin in strains with decreased susceptibility to this antibiotic. To verify this possibility, we investigated the synergism in other organisms, including different polymyxin susceptibility profiles (Table 5).

**Table 5:** Synergism of p-BthW and polymyxin B against Gram-Negative Bacterial Strains^a^

				combination (μg/mL)		
bacterial strains	main phenotype	p-BthW MIC (μg/mL)	polymyxin B MIC (μg/mL)	MIC_p-BthW_	MIC_Polymyxin B_	FIC index
*K. pneumoniae* ATCC 700603		32	1	32	1	2
*K. pneumoniae* AMKP7^27^	KPC^+^, CL S	128	0.25	8	0.06	0.3
*K. pneumoniae* AMKP4^27^	KPC^+^, CL R	512	>128	512	>128	N.D.
*K. pneumoniae* AMKP10^27^	KPC^+^, CL R	512	>128	512	>128	N.D.
*A. baumannii* ATCC 19606		32	1	2	0.12	0.2
*A. baumannii* ACI40^21^	CL S	32	0.5	<0.06	<0.06	<0.1
*A. baumannii* ACI50^21^	CL R	32	128	4	2	0.1
*E. coli* ATCC 25922		8	0.5	2	0.06	0.4
*P. aeruginosa* ATCC 27853	inducible AmpC	64	1	4	0.25	0.3

a KPC^+^, *Klebsiella pneumoniae* Carbapenemase (KPC) producing bacteria; CL R, Resistant to colistin; CL S, Susceptible to colistin. N.D. Not determined.

Polymyxin B acts as described for many AMPs: its electrostatic attraction to the negative charges of lipopolysaccharide (LPS) causes the initial interaction, displacing Ca^2+^ and Mg^2+^ ions that stabilize the outer membrane. In the case of polymyxin B, there is also a phenomenon called “self-promoted uptake”. The fatty acid part of this molecule also promotes hydrophobic interactions with LPS, which allows polymyxin to insert itself into the outer membrane, changing the permeability and leading to transient cracks that will enable the entry of various molecules, including small proteins.^28−30^

An FIC > 0.5 was found when the combination was tested against two polymyxin-resistant *K. pneumoniae* isolates. However, the FIC was lower for the polymyxin-resistant *A. baumannii* isolate. The divergent synergism results among polymyxin-resistant bacteria might be due to the different mechanisms of resistance of each isolate.^21,27,31^ These findings, therefore, indicate that polymyxin facilitates the action of p-BthW in the synergism mechanism, not the opposite.

### Membrane Depolarization

**Figure 3:**
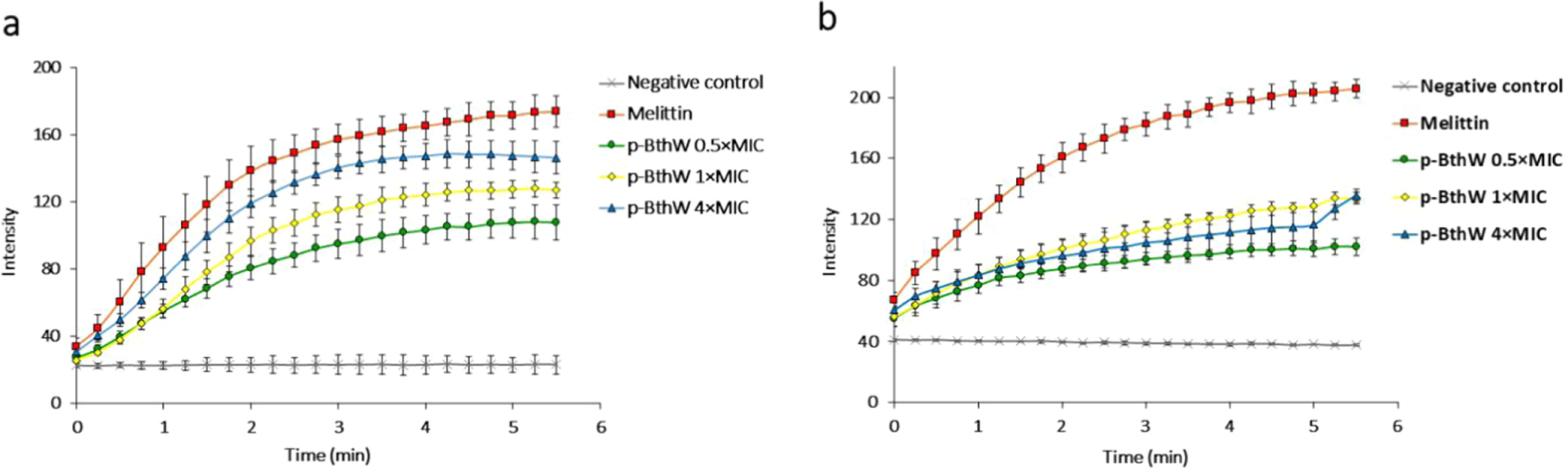
p-BthW cytoplasmic membrane depolarization for (a) *S. aureus* ATCC 25923 (MIC = 16 μg/mL) and (b) *A. baumannii* ATCC 19606 (MIC = 32 μg/mL).

The assay is based on the DISC_3_(5) fluorophore that internalizes the cells with no signal detection. A disruption in the cytoplasmic membrane (disruption, pore formation, etc.) triggers a depolarization, and the fluorophore leaves the cell, passing into the culture medium and generating a signal. This signal can then quantify with which speed and intensity a given compound causes membrane depolarization. The depolarization caused by p-BthW is represented in Figure 3.

Initially, it is possible to observe that in both *S. aureus* and *A. baumannii*, the depolarization caused by p-BthW occurs quickly within 5 min of exposure, consistent with the rapid cell death observed in the time-kill assay. In *S. aureus*, as the positive control melittin caused 100 ± 5% of depolarization and p-BthW at 4× MIC caused about 85 ± 11%, it is possible to consider both signals equivalent. In *A. baumannii*, however, the same effect is not observed. Indeed, all p-BthW concentrations tested resulted in similar depolarizations (4× MIC caused 66 ± 6%, 1× MIC caused 65 ± 6%, and 0.5× MIC caused 50 ± 8% depolarization), suggesting that there is a saturation of the peptide’s ability to cause cytoplasmic depolarization. Thus, it is possible that in Gram-negative bacteria, this peptide presents an alternative mechanism of action since the kinetics of death assay confirmed the complete death of the bacterial population at these concentrations. Similarly, the peptide (p-BthTX-I)_2_ also presented action other than exclusively in the cytoplasmic membrane for Gram-negative bacteria.^12,32^ Most of the mechanism seems to be preserved among the analogues even with the modifications made.

### Transmission Electronic Microscopy

**Figure 4:**
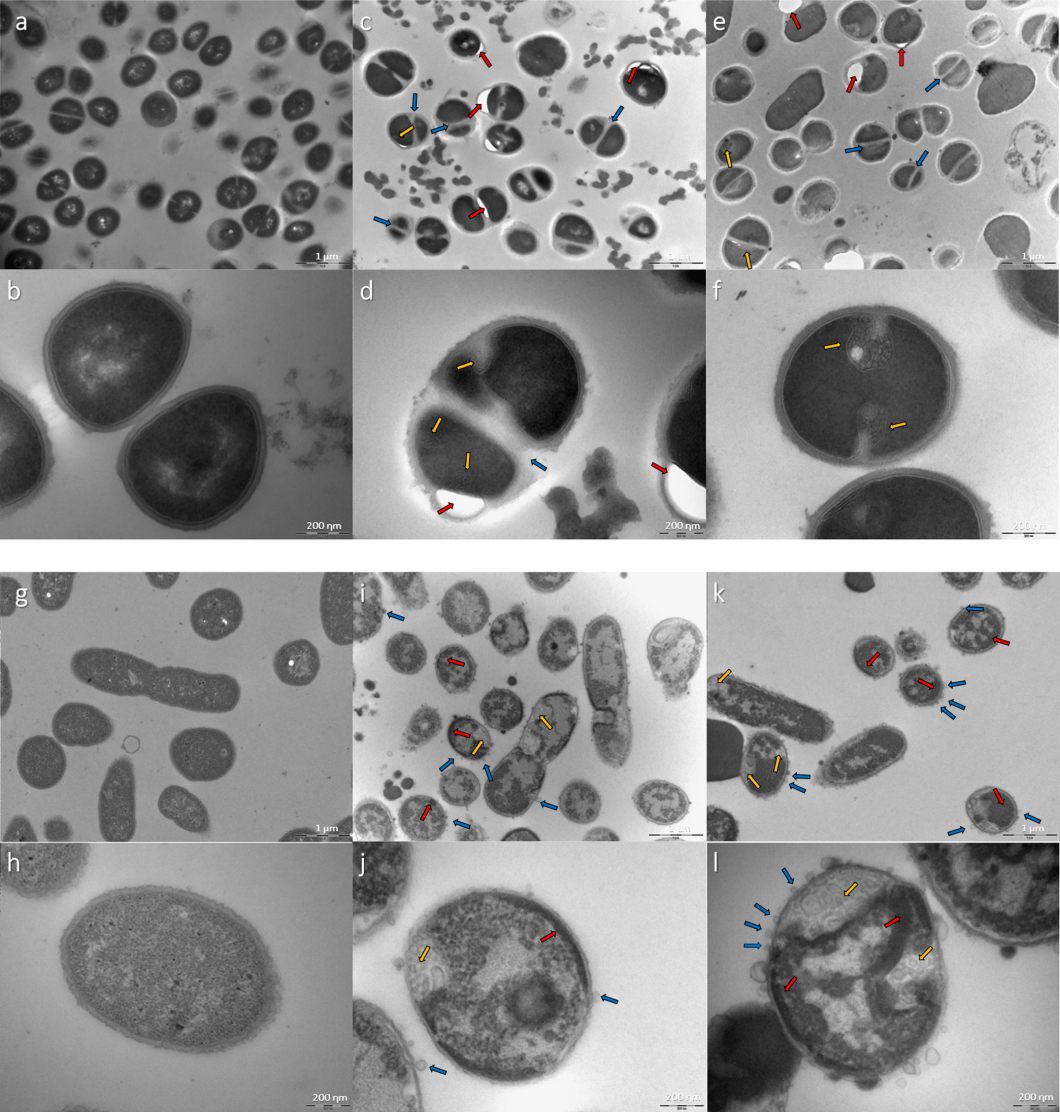
TEM images of *S. aureus* ATCC 25923 treated with p-BthW (MIC = 16 μg/mL). Red arrows point to membrane-wall displacements, blue arrows point to defective dividing septa, and yellow arrows point to membranous invaginations. (a) Nontreated cells, scale bar = 1 μm; (b) nontreated cell in detail, scale bar = 200 nm; (c) 1× MIC-treated cells, scale bar = 1 μm; (d) 1× MIC-treated cells in detail, scale bar = 200 nm; (e) 4× MIC-treated cells, scale bar = 1 μm; (f) 4× MIC-treated cells in detail, scale bar = 200 nm. TEM images of *A. baumannii* ATCC 19606 treated with p-BthW (MIC = 32 μg/mL). Red arrows point to condensed cellular contents, blue arrows point to rough and blistered surfaces, and yellow arrows point to intracellular vesicles. (g) Nontreated cells, scale bar = 1 μm; (h) nontreated cell in detail, scale bar = 200 nm; (i) 1× MIC-treated cells, scale bar = 1 μm; (j) 1× MIC-treated cells in detail, scale bar = 200 nm; (k) 4× MIC-treated cells, scale bar = 1 μm; (l) 4× MIC-treated cells in detail, scale bar = 200 nm.

To verify the mechanism of action of p-BthW, transmission electron microscopy of cells treated with this peptide (Figure 4) was performed.

Cellular debris and membranous invaginations were observed in all images of *S. aureus* treated with 1× and 4 × MIC for 10 min. Due to the rapid biocidal effect of p-BthW, these debris may come from cells that suffered damage in the first minutes of exposure. In the remaining cells, it was possible to observe defects in the division septum: asymmetric divisions, irregular septa, and more than one septum being formed simultaneously. Among the cells that were dividing, about 71 ± 15 and 67 ± 14% had some anomaly when exposed to p-BthW at 1× MIC and 4× MIC, respectively. Finally, a phenomenon in which the membrane seems to detach from the bacterial wall was observed, forming empty spaces in the form of bubbles. This damage was not observed in any of the control cells of *S. aureus* ATCC 25923, while for treated cells, it appeared with the frequency of 32 ± 7 and 17 ± 8% at 1× MIC and 4× MIC, respectively. The observed membranous invaginations in *S. aureus* have already been previously described for other AMPs, specifically as intracellular lamellar membranes or mesosomes. Usually, these mesosomes are associated with a pattern of reorganization of the cytoplasmic membrane, mainly in Gram-positive bacteria, indicating alteration or damage.^33,34^ The other damage observed (defect in the septum and membrane detachment) may indicate the action of p-BthW on the cell wall biosynthesis, which is crucial for bacterial protection, integrity, and viability. In this way, even interfering with just a single target of the machinery can disturb the entire biosynthetic apparatus, and even adjacent enzymatic machinery (such as divisome and replisome) compromises cell survival.^35^ In addition to the enzymatic machinery, another way in which the cell wall can be affected is by interfering with the substrates that form the connection between the cell wall and the cell membrane. Such an action could explain the observed detachment between the membrane and the cell wall upon p-BthW treatment. Among the targets commonly associated with the inhibition of cell wall biosynthesis, lipid II is one of the most prominent targets for several reasons: it is highly conserved among bacteria, it is easily accessed on the bacterial exterior, it offers multiple sites of interaction, and it is a nonprotein target (which makes modifications that lead to resistance more difficult).^35,36^

We cannot yet disregard other mechanisms leading to defective bacterial division, including DNA damage or damage to proteins and machinery involved in their replication. Living organisms coordinate cell division with the replication of genetic content. Bacteria, in particular, coordinate the division with the segregation of the chromosome to the poles of the cell as a way to guarantee that the septum formation in the middle of the cells would not cause damage to the DNA.^37^ Thus, bacteria have several mechanisms to verify DNA separation or damage. If there is damage to the genetic material or incorrect segregation in the daughter cells, the cell suspends division (using cytoplasmic proteins as inhibitors) until proper repair occurs.^37,38^ Thus, damage to the DNA or proteins associated with replication can interrupt the division process and formation of the septum, which may also be a valid hypothesis for the observed defects. Other AMPs that cause DNA damage and impede the process of replication and division have been reported, such as bleomycin and phleomycin, which cause breaks in the phosphodiester backbone. This DNA damage triggers a cascade of stress reactions called the stress response SOS. This SOS response affects the transcription of DNA repair genes, tolerance to genetic material damage, and cell division regulation.^29,37,38^

*A. baumannii* are coccobacilli, presenting a slightly elongated shape. The cell contents appear equally distributed, and it is possible to observe the outer membrane, cell wall, and cytoplasmic membrane uniformly in the cell. Cells treated with 1× MIC or 4× MIC have darker and lighter spots, indicating condensation of cell content. Another notable effect of treating *A. baumannii* with the peptide was the formation of vesicles, both as a direct consequence of extravasation of intracellular contents, forming bubbles that expand from the outer membrane, and what appear to be intracellular vesicles formed from the cytoplasmic membrane. There is no explanation for the observed vesicular forms, which appear to be contained within the cell and with a visible and well-defined membrane. Bacterial vesicles can form for various reasons—mainly to carry specific molecules, such as virulence, communication molecules, etc. In the case of antibiotics, three mechanisms are known to induce vesicle formation: damage to the cell envelope, inhibition of cell wall biosynthesis, and induction of SOS response.^39^ The rapid depolarization peak (less than 5 min) associated with the immediate cellular reduction demonstrated by the kinetics of death leads to the hypothesis that the membrane action is, in fact, the primary mechanism of action of p-BthW, occurring in the first minutes. However, the data indicates that secondary interactions must occur that lead to the other observed defects, including the induction of vesicle formation. These findings reiterate those described for (p-BthTX-I)_2_ since the hypothesis is that this peptide has late effects other than just membrane action. The internal vesicular formation caused by p-BthW resembles an apoptosis-like mechanism, a mode of action already deliberated for (p-BthTX-I)_2_^12,32^

### In Vitro Directed Evolution

To assess the possibility of obtaining isolates with reduced sensitivity to p-BthW and to study possible mutated genes that could indicate the mechanism of action of this peptide, an in vitro directed evolution was performed with sub-inhibitory concentrations of the peptide. The same was done with control antibiotics in parallel: ciprofloxacin, representing an antibiotic with an intracellular target (prevents DNA replication by inhibiting bacterial topoisomerases and DNA-gyrase), and polymyxin B or daptomycin, representing an action on the outer membrane and cell wall plus membrane, respectively.

**Figure 5:**
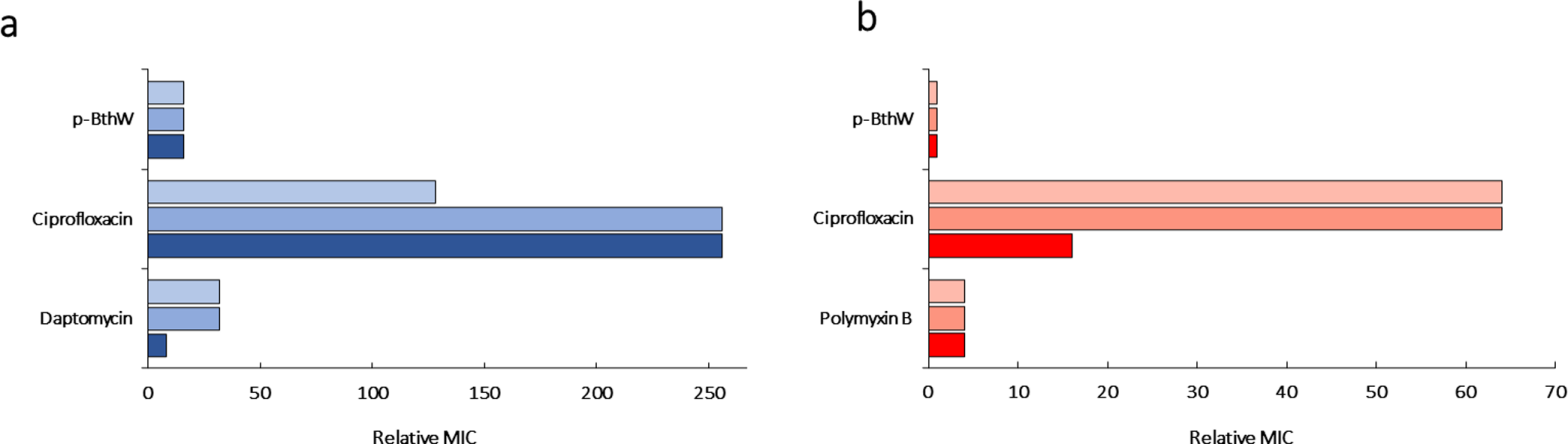
Relative MIC increase for each 30-day exposure, including exposure with p-BthW, each biological replicate represented in a bar. (a) *S. aureus* ATCC 25923 (b) *A. baumannii* ATCC 19606.

The increase in MIC of p-BthW and other antibiotics for Gram-positive and Gram-negative bacteria after 30 days of in vitro directed evolution is represented in Figure 5.

For *S. aureus*, while the MIC for p-BthW increased from 16 to 256 μg/mL (16-fold increase), ciprofloxacin caused an increase from 0.25 to 32 and 64 μg/mL (128- and 256-fold increase), and daptomycin from 0.125 to 1 and 4 μg/mL (8- and 32-fold increase). For *A. baumannii*, the MIC for p-BthW stayed stable at 32 μg/mL, ciprofloxacin selection resulted in a MIC jump from 1 to 16 and 64 μg/mL (16- and 64- fold increase), and for Polymyxin B from 0.25 to 1 μg/mL (4-fold increase).

Genome comparison of strains before and after the directed evolution was performed to understand which genetic changes could have increased the p-BthW MIC in *S. aureus*. Single-nucleotide polymorphisms found are in Table 6.

**Table 6:** Genomic Comparison of the Initial and Final Strains of the *S. aureus* ATCC 25923 In Vitro Selected by p-BthW

replicate	sequencing coverage^a^	N50	mutation	change	altered protein
A	90×	6925	331G>A	Ala111Thr	hypothetical protein
B	111×	11159	394_395InsACGCTGATGTTGTTGAATATGAA	frameshift mutation	fibronectin binding precursor (FnbA)
C	105×	4930	2678T>C	Leu893Ser	clumping factor A (ClfA)
			257T>C	Phe86Ser	β subunit of α-ketoacid dehydrogenase

a Coverages of the initial strains were 148×, 97×, and 112×, respectively.

Regardless of the mode of action, the in vitro selection forecasts a remarkable perspective for resistance occurrence for this molecule. The concern with antibiotics and their propensity to select resistant strains has reached worldwide importance and is increasingly emphasized in the search for new antimicrobial molecules. This concern occurs with antibiotics and even biocides used as disinfectants in hospital or domestic environments. For example, the European regulation on biocidal products has required the manufacturer to communicate whether the product can lead to active substance resistance or even cross-resistance.^40^ The unchanged MIC for *A. baumannii* in the direct evolution is particularly relevant—antibiotics that act on the membrane or cell wall (such as daptomycin and polymyxin) are less likely to develop resistance. As nonprotein targets, alterations that lead to a change in affinity have a higher metabolic cost than a mutation in a specific protein.^41^

On the other hand, for *S. aureus*, where there was a selection of resistant isolates, comparing the initial and final strains may indicate which mutations led to an increase in the MIC. Genes that encode the target of p-BthW, proteins of its entry pathways into the cell, or even efflux pumps, can be more easily mutated in Gram-positive bacteria, increasing the MIC, than in Gram-negative bacteria, in which activity remained stable. Thus, the genome sequencing of the strains allows this comparison and may be essential to verify the mechanisms of resistance and, consequently, one more way to verify the hypotheses raised about the mechanism of action of p-BthW.

In selected strain A, there was a mutation in a hypothetical protein that consists of a series of serine and aspartate amino acids repeated in succession, occasionally intercalated by glutamate or alanine. This protein seems connected to the clumping factor A (ClfA), which also mutated in experiment C. In the ClfA protein, this same repeating sequence can be observed. Thus, the alterations involving both mutations might be related. The *clfA* gene encodes a high-molecular-weight protein anchored to the cell wall. This protein promotes adhesion by binding fibrinogen and causing the plasma clumps seen in *S. aureus* infections. Thus, this protein is intrinsically related to the virulence of the organism. Under physiological conditions, the aspartate side chains will have a negative charge due to acid deprotonation, and the interspersing serine residues are hydrophilic and highly dispersed. Thus, the repulsion between aspartates added to serine interdispersion generates an electrostatic driving force that extends the protein away from the cell surface.^42−44^ The charges contained in this serine-aspartate repeat region are sufficient for the electrostatic attraction of the positively charged antimicrobial peptide. Interestingly, the mutation did not occur in the coding region of the anchor, nor in the negative amino acids, but rather in an alanine that intercalates the serine-aspartate repeats. As the structure of this portion of the serine-aspartate repeat protein is mainly random coil, it is unknown precisely what alteration this mutation caused. The FnbA protein, which suffered mutation in the selected strain B, is a protein anchored in the membrane and cell wall, contains the LPXTG motif, has the function of adhesion and internalization by host cells, and is capable of binding to soluble and immobilized fibronectin, invading cells even without additional factors.^45,46^ FnbA is also capable of binding elastin and fibrinogen. Thus, it acts not only on the infection but also on the persistence of such infection. The insertion mutation occurred in the A portion of the protein, which is the active site of FnbA. Mutations in this region lead to loss of function: there is no longer the ability to bind fibrinogen and elastin.^47^ There is no reported correlation between such proteins and the action of antimicrobial peptides nor the mechanism of action of other antibiotics.

As for the last mutation in one of the replicates, the α-ketoacid dehydrogenase complex protein is homologous to branched-chain α-ketoacid dehydrogenase, with 50% identity at 100% coverage. This protein is part of the cycle of reactions that catabolizes branched-chain amino acids such as valine, leucine, and isoleucine. In addition, this protein has also been linked to membrane fluidity in *S. aureus*.^48^ Fatty acids originating from branched and unsaturated chains are considered more fluid, while single or saturated chains attribute more rigid characteristics to the membrane.^49^ The knockout of the gene that encodes α-ketoacid dehydrogenase branched-chain amino acids showed in *Bacillus subtilis* that the lack of activity of this protein leads to greater rigidity in the membrane due to the greater use of single-chain amino acids. AMPs may prefer microdomains of the branched-chain membrane for insertion and action.^28,29^

The α-ketoacid dehydrogenase β-subunit enzyme has a single AcoB domain, a conserved domain in the family, a component related to energy production and conversion.^50,51^ Thus, p-BthW may act on cellular respiration or catabolism. Finally, these two hypotheses about the mutation may be related: respiratory metabolism and cell growth rate depend on membrane fluidity. The more fluid lipids rigidly control the respiratory mechanism, probably due to the electron transport chain that depends on the diffusion of proteins and electron transport enzymes. Thus, the electron transport chain depends on membrane diffusion, which may restrict the evolution of the composition of these membranes.^52^

## Conclusions

Dimerization has already proved to be an efficient strategy for increasing the antimicrobial activity of peptides derived from p-BthTX-I. In the peptide used in this work, the dimerization in the C-terminal portion and the use of the amino acid tryptophan optimized the activity, mainly in Gram-negative bacteria. This peptide’s rapid and broad-spectrum action was beneficial despite showing a high level of cytotoxicity to differentiated macrophages. The mechanism of action of p-BthW involves not only activity on the cytoplasmic membrane but appears to be associated with activity on cell wall precursors. Directed evolution also indicates that this molecule has a low tendency to develop resistance, a highly desired characteristic for new antimicrobials. Overall, despite the results reported, p-BthW still requires many additional tests to be considered a candidate for systemic use.

## Methods

### Peptide Synthesis

Solid-phase peptide synthesis^53^ was performed manually using the 9-fluorenylmethyloxycarbonyl (Fmoc) protocol.^54^ Deprotection of the Fmoc group was performed in 20% 4-methylpiperidine in *N*,*N*-dimethylformamide (DMF) for 1 and 20 min. In all cases, the amino acids were coupled in excess (2×) using *N*,*N*′-diisopropylcarbodiimide/*N*-hydroxybenzotriazole in 50% (v/v) dichloromethane/DMF solution. After 2 h of reaction, positive coupling was assessed using the ninhydrin test.^55^ Resin cleavage and removal of side-chain-protecting groups were performed for 2 h at a ratio of 10 mL/g resin, using 95% trifluoroacetic acid (TFA), 2.5% triisopropylsilane, and 2.5% water. Thus, the crude peptides were precipitated with ethyl ether and separated from the soluble nonpeptide material by centrifugation. The peptide was extracted with a solution containing 0.045% (v/v) TFA in water and lyophilized.

### Purification and Characterization of Peptides by HPLC

Purification of the synthetic peptides was performed in a semipreparative mode using an HPLC System LC20-AT (Shimadzu) in a C18 reversed-phase (2.1 × 25 cm^2^) semipreparative column (Phenomenex). The purity was determined on a chromatographer LC-10ATVP (Shimadzu) using a C18 reversed-phase (0.46 × 25 cm^2^) analytical column (Kromasil). Solvents were 0.045% TFA in ultrapure water (Solvent A) and 0.036% TFA in acetonitrile (Solvent B). The peptides were obtained with a high purity level (above 95%).

### Mass Spectrometry

In order to confirm peptide identity, electrospray mass spectrometry was obtained from an LCQ FLEET (Thermo Scientific) by direct injection in positive detection mode.

### Antimicrobial Susceptibility Evaluations

#### MIC and MBC Determination

The microdilution method outlined by the Clinical and Laboratory Standards Institute^56^ was utilized to determine the minimum inhibitory concentration (MIC) of p-BthW. The peptide was diluted in cation-adjusted Mueller-Hinton (CAMH) broth (BD, East Rutherford, NJ) over a concentration range of 512–0.06 μg/mL. In a final 5 × 10^5^ CFU/mL concentration, a bacterial inoculum was added to the peptide. Tables S1 and S2 provide descriptions of the bacteria used in this study. After incubation at 35 °C for 22 h, the MIC was determined as the lowest concentration that inhibited visible microbial growth. All assays were conducted in triplicate using polystyrene U-bottom microplates with minimal protein binding. The negative control utilized bacterial growth without any antimicrobial.

A total of 100 μL from each well in the MIC assay was subcultured on CAMH agar plates and incubated at 35 °C for 24 h to determine the minimum bactericidal concentration (MBC). The MBC was the lowest peptide concentration with no visible growth on the plate.

#### Time-Kill Assay

The experiments were conducted under the guidelines recommended by the CLSI.^56^ p-BthW activity over time was assessed against two species known to cause a high burden of MDR infections: *S. aureus* ATCC 25923 and SA43 strains,^19^ as well as *A. baumannii* ATCC 19606 and ACI50 strains.^27^ Inoculum containing 6 × 10^5^ CFU/mL were exposed to the peptide or antibiotics concentrations at 0.5×, 1×, 2×, and 4× the MIC. Aliquots (20 μL) were collected at 0, 15, and 30 min, as well as 1, 2, 4, 8, 12, and 24 h, and serially diluted (1:10) in 0.85% sterilized saline. The microdrop technique was then employed to cultivate the samples on Brain and Heart Infusion (BHI) agar, which were incubated at 37 °C for 24 h. Later, colonies were counted, with bacterial growth without the peptide serving as the negative control, while daptomycin and polymyxin B were used as positive controls. Technical sextuplets were used, and the experiment was performed in biological duplicate. The detection limit for the assay was 10^2^ CFU/mL.

#### Post-Antibiotic Effect

Post-antibiotic effects were evaluated following the protocol of Saravolatz et al.,^23^ using the same bacterial strains as in the time-kill assay. A bacterial inoculum of 6 × 10^7^ CFU/mL was added to 10 mL of MHCA containing 0.5× and 1× the MIC of the peptide, and the tubes were homogenized and incubated at 37 °C for 10 min before being centrifuged at 3000*g* for 10 min at room temperature. The supernatant was discarded, and the bacteria were resuspended in 10 mL of fresh MHCA at 37 °C. An inoculum (20 μL) was taken hourly, and the same method used in the time-kill assay was used for CFU quantification. The post-antibiotic effect (PAE) was calculated using the equation PAE = *T* – *C*, where *T* represents the time for the treated sample to increase by 1 log and *C* represents the time for the growth control to increase by 1 log. The negative control was bacterial growth without peptides, while daptomycin and polymyxin B were positive controls. The assay was performed in biological duplicates and technical sextuplets, and the concentrations and strains treated were compared using ANOVA with a significance threshold of *p* < 0.05.

#### Synergism with Antibiotics

Synergism was assessed using checkerboard analysis, using the method described in the MIC and MBC Determination section, by diluting p-BthW (compound A) horizontally and antibiotics (compound B) vertically in the same microplate. The ATCC strains were used to evaluate the combination of p-BthW with commercial antibiotics of different classes: Ciprofloxacin, tobramycin, vancomycin, ampicillin, and imipenem were tested against *S. aureus* ATCC 25923 and *A. baumannii* ATCC 19606, along with the peptides. If synergism was observed for a specific combination, this combination would be tested against different species, ATCC and clinical strains. Daptomycin and polymyxin B were tested against Gram-positive and Gram-negative bacterial strains, respectively. The fractional inhibitory concentration (FIC) was calculated using the formula:
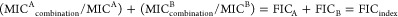


Synergism was defined as an FIC < 0.5.^57^ Assays were conducted in biological and technical triplicate.

#### Mode of Action Assays

##### Membrane Depolarization

The cytoplasmic membrane depolarization assay was conducted using 3,3’-dipropylthiadicarbocyanine iodide, DISC3(5), following the protocol described by a previous study^58^ using *S. aureus* ATCC 25923 and *A. baumannii* ATCC 19606. Bacterial colonies were grown in MHCA with agitation at 37 °C until the mid log phase (between 4 and 6 h). The bacterial culture was centrifuged at 4000 rpm for 10 min at room temperature. The supernatant was discarded, and the cells were resuspended in a respiration buffer (5 mM HEPES and 20 mM glucose, pH 7.4). The cells were centrifuged, suspended in fresh buffer, and adjusted to OD_600_ = 0.05 using SpectraMax M5 (Molecular Devices, CA). A final concentration of 0.2 μM DISC3(5) was added to the cells, and 0.05 mM EDTA was only added for *A. baumannii*. The cells were then incubated with DISC3(5) for 1 h at room temperature. After incubation, 200 μL of the cell suspension was added to each well of a matte-black, flat-bottom microplate. To each well, 2 μL of the peptide was added at a final concentration of 4×, 1×, or 0.5× MIC. A positive melittin control was included at a final concentration of 10 mg/mL (100% depolarization, always compared to depolarization peak). Assays were performed in biological and technical triplicate. The microplate was read on a SpectraMax M5 (Molecular Devices, CA) in fluorescence mode, excitation at 622 nm, and emission at 670 nm for 5 min.

##### Transmission Electronic Microscopy

Transmission electron microscopy (TEM) was used to observe *S. aureus* ATCC 25923 and *A. baumannii* ATCC 19606 cells untreated or treated with p-BthW as described previously.^59^ Isolated colonies were grown in CAMH under agitation at 37 °C until the mid log phase. Next, 30 mL was adjusted to OD_600_ = 0.05 using a SpectraMax M5 (Molecular Devices, CA) in CAMH and treated with the peptide at 0.5×, 1×, or 4× MIC. After incubation at 37 °C for 10 min, the bacteria were centrifuged thrice at 3000*g* for 10 min and washed with PBS. The bacteria were then resuspended in PBS with 3% glutaraldehyde and incubated at 4 °C for 2 h, followed by another centrifugation step. Cells were resuspended in PBS and fixed with 3% glutaraldehyde for 2 h at 0 °C, then fixated with osmium tetroxide for 2 h at 4 °C. The samples were then washed and dehydrated using increasing concentrations of ethanol. After the final ethanol wash, the cells were resuspended in propylene oxide and centrifuged twice. The oxide was removed, and the material was deposited on epoxy resin and stirred overnight. Ultrafine cuts were made using an ultramicrotome. The sections were analyzed using a JEOL 100CXII microscope (Japan). Ten images were captured randomly at 20,000× magnification to compare the treated and untreated samples. The samples were prepared in biological duplicates and compared using ANOVA for quantitative analysis.

### Toxicity Assays

#### Hemolytic Activity

The protocol described by Castro et al.^60^ was used to determine the hemolytic activity of p-BthW. This study was approved by the Ethics Committee of the Federal University of São Carlos (CAAE 52231421.7.0000.5504). Blood from medication-free human volunteers was collected in tubes containing ethylenediaminetetraacetic acid (EDTA) and washed thrice with phosphate-buffered saline (PBS). The precipitated cells were resuspended in 1% PBS. Erythrocytes were exposed to the peptide for 1 h at 37 °C at concentrations ranging from 512 to 0.06 μg/mL. Triton X-100 1% served as a positive control for hemolysis. Following incubation, the microplate was centrifuged, and the hemolytic rate was determined by measuring the absorbance of the supernatant at 405 nm. The percentage of hemolysis was calculated using the formula:
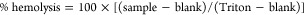


The HC_50_ value was calculated as the peptide concentration required for 50% hemolysis. The assays were performed in biological and technical triplicate. HC_50_ was calculated using logarithmic regression with GraphPad Prism software.

#### Cytotoxicity and Selectivity Index

Cytotoxicity assays^26^ were conducted using THP-1 (differentiated human macrophages) and HFF-1 (human fibroblasts) cell lines. The cells were seeded in 96-well plates and allowed to grow for 24 h at 37 °C before exposure to p-BthW. The concentrations used ranged from 0.06 μg/mL to 512 μg/mL, as performed for MIC determination. After 24 h of incubation, cell viability was evaluated using the MTS assay, which involves the addition of MTS reagent and subsequent measurement of absorbance at 490 nm using a SpectraMax 384 spectrophotometer (Sunnyvale, CA). Doxorubicin was used as a positive control, and all assays were performed in triplicate. The concentration that reduced cell viability by 50% (CC_50_) was calculated by data fitting using GraphPad Prism 8.0 software. The percentage of nonviable cells was determined and compared to the negative control wells, which were set to 100% growth. The selectivity index (SI) was calculated as the ratio between CC_50_ and MIC_50_.

### Resistance Assay

#### In Vitro Directed Evolution

The in vitro directed evolution of *S. aureus* ATCC 25923 and *A. baumannii* ATCC 19606 was conducted in triplicate, guided by the presence of p-BthW and antibiotics, following the protocol described by Jahnsen et al.^58^ Ciprofloxacin, daptomycin, and polymyxin B were used to treat the cells in addition to the peptide. The MIC of the peptides and antibiotics was determined as described in the Mass Spectrometry section. For the MIC reading, the absorbance at 600 nm was measured using a Spectramax M5 (Molecular Devices, CA). If the inhibition was 50% or more, the content of the well was diluted 1:20 and used as a new bacterial inoculum for a new MIC microplate. This process was repeated daily for 30 days. After 30 days, the final lines were passaged three times in CAMH free of antibiotics or peptides for stabilization. The selected strains had their MIC evaluated for other antibiotics to check for cross-resistance.

#### Genome Sequencing

Following directed evolution, the isolates displaying reduced susceptibility (compared to the wild-type strains and initial MIC) were subjected to DNA extraction for genome sequencing. The QIAGEN Dneasy Blood and Tissue Kit was utilized for the extraction following the manufacturer’s instructions. Library preparation for Illumina sequencing was performed using the Nextera XT DNA Library Preparation Kit, as specified by the manufacturer. The quality and quantity of each sample library were measured using the TapeStation instrument from Agilent Technologies. The genomes were sequenced using an Illumina MiSeq sequencer as 2 × 250 bp reads, with a minimum depth of coverage of 126× (ranging from 126× to 242×). The sequence reads were assembled de novo, and variant detection was carried out using the CLC Genomics Workbench (CLC Bio, Cambridge, MA). The accession numbers for this whole-genome shotgun project have been deposited at DDBJ/ENA/GenBank under the accession numbers: JAKSZW000000000, JAKSZX000000000, JAKSZY000000000, ALLNU000000000, JALLNS000000000, JAKWBG000000000.

## Data Availability

The data is either available in the paper or in the online repository accessed through the link https://drive.google.com/drive/folders/1TqNufGnxE1fHZKZQaCm8KQg3IuCApBVc?usp=sharing.

## Supporting Information Available

Representative scheme of peptide p-BthW, with cationic amino acids colored in red and hydrophobic amino acids colored in blue (Figure S1); MIC, MBC, and SI of p-BthW against Gram-positive bacteria (Table S1); MIC, MBC, and SI of p-BthW against Gram-negative bacteria (Table S2); and synergism for p-BthW in *S. aureus* ATCC 25923 (MIC = 16 mg/L) (Table S3) (PDF)

## Notes

This study was approved by the Ethics Committee of the Federal University of São Carlos (CAAE 52231421.7.0000.5504).
