# Supplementary material for: Optimizing Bothropstoxin-I-Derived Peptides: Exploring the Antibacterial Potential of p-BthW
Source: ACS Omega. 2024 May 22;9(22):23662–74. doi: 10.1021/acsomega.4c01303 (PMC11154919; doi:10.1021/acsomega.4c01303)
Supplement: Supplementary file 1 — ao4c01303_si_001.pdf [file ao4c01303_si_001.pdf]

Supporting Information

Optimizing Bothropstoxin-I-Derived Peptides: Exploring the Antibacterial Potential of p-BthW

Gabriela Marinho Righetto[1], Norival Alves Santos-Filho[2], Letícia Oliveira Catarin Nunes[2], Camille André[3], Julia Medeiros Souza[4], Adriano Defini Andricopulo[4], Paulo José Martins Bispo[3], Eduardo Maffud Cilli[2*], Ilana Lopes Baratella da Cunha Camargo[1*]

1 Laboratory of Molecular Epidemiology and Microbiology, Department of Physics and Interdisciplinary Science, University of Sao Paulo, 13563-120, São Carlos, Brazil; gmrighetto@gmail.com (G.M.R.), ilanacamargo@ifsc.usp.br (I.L.B.C.C.)
2 São Paulo State University Institute of Chemistry, Department of Biochemistry and Organic Chemistry, 14800-060, Araraquara, Brazil; leticia.catarin@unesp.br (L.O.C.N.), norival.santos-filho@unesp.br (N.A.S-F), eduardo.cilli@unesp.br (E.M.C.)
3 Infectious Disease Institute, Department of Ophthalmology, Massachusetts Eye and Ear, Harvard Medical School, MA 02115, Boston, USA; Paulo_Bispo@meei.harvard.edu (P.J.M.B.), candre2@meei.harvard.edu (C.A.)
4 Laboratory of Medicinal and Computational Chemistry, Department of Physics and Interdisciplinary Science, University of Sao Paulo, 13563-120, São Carlos, Brazil; juliamedeiros1612@gmail.com (J.M.S), aandrico@ifsc.usp.br (A.D.A.)

* Correspondence: I.L.B.C.C. ilanacamargo@ifsc.usp.br
E.M.C. eduardo.cilli@unesp.br

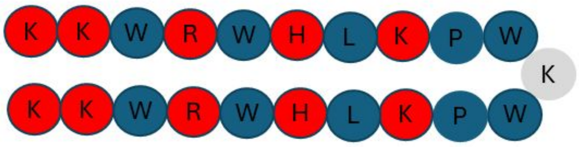

Figure S1. Representative scheme of peptide p-BthW, with cationic aminoacids colored in red and hydrophobic aminoacids colored in blue.

Table S1. MIC, MBC, and SI of p-BthW against gram-positive bacteria

Species	Strains	Description	MIC		MBC		SI	
			µg/mL	µM	µg/mL	µM	THP-1	HFF-1
S. epidermidis	ATCC 35984	Clinical strain, catheter isolate. Strong biofilm former	8	2.6	16	5.3	7.6	32.2
	ATCC 12228		4	1.3	16	5.3	15.2	64.4
S. aureus	ATCC 25923	Clinical strain.	16	5.3	32	10.5	3.8	16.1
	SA16	Clinical strain. MRSA, ST5-SCC*mec*II	16	5.3	32	10.5	3.8	16.1
	SA88*[15]	Clinical strain. MRSA, ST5-SCC*mec*II + h-DNSSA	16	5.3	32	10.5	3.8	16.1
	SA43 [15]	Clinical strain. MRSA, ST105-SCC*mec*II, TIG S	16	5.3	32	10.5	3.8	16.1
	SA43 B2 [15]	*In vitro* selected MRSA, ST105-SCC*mec*II, TIG S	16	5.3	16	5.3	3.8	16.1
	SA43 B7 [15]	*In vitro* selected MRSA, ST105-SCC*mec*II, TIG MIC increased MepA super expressed (*mepR* mutation)	32	10.5	32	10.5	1.9	8.1
	Mu50 [56]	MRSA, VISA, ST5	8	2.6	32	10.5	7.6	32.2
	ATCC 8095	Food isolate. Strong biofilm former	16	5.3	32	10.5	3.8	16.1
E. faecalis	VRE 109 [57]	Clinical strain, ST103*, vanA* TIG S, VAN R	64	21.1	>256	>84.2	0.9	4
	VRE 109 C42 [57]	*In vitro* selected. ST103, *vanA* TIG R, VAN R	64	21.1	256	84.2	0.9	4
	VRE 80 [57]	Clinical strain. ST103, *vanA* TIG R, VAN R,	64	21.1	256	84.2	0.9	4
	V583	ST 6, *vanB*, VAN R, CN R	128	42.1	256	84.2	0.5	2
	RPEfs1*	Clinical strain. AMP S, CIP R, ERY R, LNZ S, MXF R, TEC S, TIG S, VAN S	128	42.1	256	84.2	0.5	2
	RPEfs2*	Clinical strain. AMP S, CIP S, ERY I, LNZ S, MXF S, TEC S, TIG S, VAN S	128	42.1	256	84.2	0.5	2
	RPEfs3*	Clinical strain. AMP S, CIP R, ERY R, LNZ S, MXF R, TEC R, TIG S, VAN R	64	21.1	256	84.2	0.9	4

Species	Strains	Description	MIC		MBC		SI	
			µg/mL	µM	µg/mL	µM	THP-1	HFF-1
	RPEfs4*	Clinical strain. AMP S, CIP R, ERY R, LNZ S, MXF S, TEC S, TIG S, VAN S	64	21.1	256	84.2	0.9	4
	RPEfs5*	Clinical strain. AMP S, CIP S, DAP S, LNZ S, NIT S, TEC S, TET R, VAN S	8	2.6	32	10.5	7.6	32.2
	ATCC 29212	Clinical strain, urine isolate	32	10.5	128	42.1	1.9	8.1
E. faecium	VRE 16 [58]	Clinical strain. ST412, *vanA*, VAN R	32	10.5	64	21.1	1.9	8.1
	HBSJRP18 [59]	Clinical strain. ST412, DAP hypersusceptible (*lafB**)	32	10.5	64	21.1	1.9	8.1
	HBSJRP18 2.7 [59]	*In vitro* selected. DAP S (*lafB* revertant)	32	10.5	64	21.1	1.9	8.1
	HBSJRP18 3.6 [59]	*In vitro* selected. DAP R (*dak**)	32	10.5	64	21.1	1.9	8.1
	HBSJRP7 [59]	Clinical strain, muscle biopsy isolate ST896, *ermB, msrC, tetL, tetM, vanA*. DAP R, LNZ S, TED S, TEC R, VAN R	8	2.6	32	10.5	7.6	32.2
	HBSJRP13 [59]	Clinical strain, bronchus alveolar lavage isolate ST896, *ermB, msrC, tetL, tetM, vanA.* DAP S, LNZ S, TED S, TEC R, VAN R	16	5.3	64	21.1	3.8	16.1
	HBSJRP14 [59]	Clinical strain, urine isolate. *vanA.* DAP S, LNZ S, TED S, TEC R, VAN R	8	2.6	32	10.5	7.6	32.2
	HBSJRP23 [59]	Clinical strain, urine isolate. DAP S, LNZ S, TED S, TEC S, VAN S	8	2.6	32	10.5	7.6	32.2
	HBSJRP11 [59]	Clinical strain, urine isolate DAP S, LNZ S, TED S, TEC S, VAN S	8	2.6	32	10.5	7.6	32.2
	ATCC 700221	Human feces isolate. *vanA*, VAN R	8	2.6	32	10.5	7.6	32.2

* Ilana Camargo, LEMiMo.

American Type Culture Collection (ATCC). Sequence type (ST). Contains vancomycin resistance element VanA (vanA), methicillin-resistant Staphylococcus aureus (MRSA); S. aureus with intermediate vancomycin resistance (VISA); R, resistant; S, sensitive, I, intermediate; Amikacin (AK), Amoxicillin-clavulanate (AMC), Ampicillin (AMP), Ampicillin-sulbactam (SAM); Aztreonam (ATM), Cefepime (FEP), Cefotaxime (CTX), Cefoxitin (FOX), Cefpodoxime (CPD), Ceftazidime (CAZ), Ceftriaxone (CRO), Cefuroxime (CXM), Ciprofloxacin (CIP), Chloramphenicol (CHL), Colistin (CL), Daptomycin (DAP), Erythromycin (ERY), Ertapenem (ETP), Gentamicin (CN), Imipenem (IMI), Linezolid (LNZ), Meropenem (MEM), Moxifloxacin (MXF), Nitrofurantoin (NIT), Piperacillin (PIP), Piperacillin-tazobactam (PTZ), Polymyxin B (PB), Tedizolide (TED), Teicoplanin (TEC), Tetracycline (TET), Tigecycline (TIG), Trimethoprim-sulfamethoxazole (SXT), Vancomycin (VAN)

Table S2. MIC, MBC, and SI of p-BthW against gram-negative bacteria

Species	Strains	Description	MIC		MBC		SI	
			µg/mL	µM	µg/mL	µM	THP-1	HFF-1
K. pneumoniae	ATCC 700603	Clinical strain, urine isolate. *bla*$_{KPC}$-, *bla*$_{SHV-18}$ + AMP R, ATM R, FOX R, CPD R, CAZ R, CHL R, PIP R, TET R	32	10.5	128	42.1	1.9	8.1
	ATCC BAA1705	Clinical strain, urine isolate. *bla*$_{KPC}$+	32	10.5	128	42.1	1.9	8.1
	BHKPC50*	Clinical strain, urine isolate. AK R, AMP R, SAM R, FEP R, FOX R, CAZ R, CRO R, CXM R, CIP R, CL R, ETP R, CN R, IMI R, MEM R, PTZ R, TIG R	128	42.1	512	168.4	0.5	2
	RPKp01*	Clinical strain, urine isolate. AK S, AMP S, SAM R, FEP R, FOX R, CAZ R, CRO R, CXM R, CIP R, CL S, ETP R, CN S, IMI R, MEM R, PTZ R, TIG R	64	21.1	128	42.1	0.9	4
	RPKp02*	Clinical strain, rectal swab isolate. AK I, AMP R, SAM R, FEP R, FOX R, CAZ R, CRO R, CXM R, CIP R, CL R, ETP R, CN S, IMI R, MEM R, PTZ R, TIG I	128	42.1	>512	>168.4	0.5	2
	RPKp09*	Clinical strain, surgical wound isolate. AK S, AMC R, AMP R, FEP R, FOX R, CAZ R, CRO R, CXM R, CIP S, CL S, ETP S, CN S, IMI R, MEM R, PTZ R, TIG R,	32	10.5	128	42.1	1.9	8.1
	RPKp18*	Clinical strain, blood isolate. AK S, AMP R, SAM R, FEP R, FOX R, CAZ R, CRO R, CXM R, CIP S, CL R, ETP R, CN S, IMI R, MEM R, PTZ R, TIG S	64	21.1	256	84.2	0.9	4
	NDM-1 (2146)**	*bla*$_{NDM}$	32	10.5	>128	>42.1	1.9	8.1
	AMKP4 [21]	Clinical strain. *bla*$_{KPC-2}$ CL R, ETP R, IMI R, MEM R, PB R, TIG S	512	168.4	>512	>168.4	0.1	0.5
	AMKP7 [21]	Clinical strain. *bla*$_{KPC-2}$ CL S, ETP R, IMI R, MEM R, PB S, TIG S	128	42.1	256	84.2	0.5	2
	AMKP10 [21]	Clinical strain. ST2306, *bla*$_{KPC-2}$, *bla*$_{CTX-M8}$, *bla*$_{SHV-11}$, *tetA, aph(3')-Ia, catB, aac(6')Ib-cr, fosA*, *bla*$_{CTX-M15}$, *oqxab, qnrS1, sul1*, *bla*$_{OXA-1}$, *aadA2, dfrA12, mph(A), mgr*B	256	84.2	>512	>168.4	0.2	1

Species	Strains	Description	MIC		MBC		SI	
			µg/mL	µM	µg/mL	µM	THP-1	HFF-1
		AK S, AMP R, SAM R, FEP R, FOX R, CAZ R, CRO R, CXM R, CIP R, CL R, ETP R, CN S, IMI R, MEM R, PB R, TIG S						
E. coli	ATCC 25922		32	10.5	64	21.1	1.9	8.1
	ATCC 35218	Canine isolate. *bla*$_{TEM-1}$	32	10.5	64	21.1	1.9	8.1
	RPEc01*	Clinical strain, urine isolate. AK R, AMP R, SAM R, FEP R, FOX R, CAZ R, CRO R, CXM R, CIP S, CL S, ETP R, CN R, IMI R, MEM R, PTZ R, TIG S	32	10.5	>128	>42.1	1.9	8.1
	BHKPC10*	Clinical strain, urine isolate. AK S, AMC R, FEP R, CAZ R, CIP S, CN S, MEM R, NIT S, SXT S.	32	10.5	64	21.1	1.9	8.1
	AMEc8*	Clinical strain, urine isolate. AK S, AMP R, SAM R, FEP R, FOX R, CAZ R, CRO R, CXM R, CIP R, CL S, ETP R, CN R, IMI S, MEM S, PTZ R, TIG I	16	5.3	16	5.3	3.8	16.1
	AMEc49*	Clinical strain, cranial subdural empyema isolate. AK S, AMP R, SAM I, FEP S, FOX S, CAZ S, CRO R, CXM R, CIP S, CL S, MEM S, PTZ S, TIG S	16	5.3	>64	>21.1	3.8	16.1
	AMEc60*	Clinical strain, tracheal secretion isolate. AK S, AMP R, SAM R, FEP R, FOX S, CAZ R, CRO R, CXM R, CIP R, CL S, MEM S, PTZ S, TIG S	32	10.5	64	21.1	1.9	8.1
A. baumannii	ATCC 19606	Clinical strain, urine isolate.	32	10.5	64	21.1	1.9	8.1
	ACI50 [16]	Clinical strain. AK R, SAM R, FEP R, CTX R, CAZ R, CRO R, CIP R, CL R, CN R, IMI R, MEM R, PTZ R PB R, TET I, TIG R, SXT R	32	10.5	32	10.5	1.9	8.1
	ACI44 [16]	Clinical strain. AK R, SAM R, FEP R, CTX R, CAZ R, CRO R, CIP R, CL S, CN R, IMI R, MEM R, PTZ R, PB S, TET R, TIG R, SXT R	32	10.5	32	10.5	1.9	8.1
	ACI51 [16]	Clinical strain. AK R, SAM R, FEP R, CTX R, CAZ R, CRO R, CIP R, CL R, CN R, IMI R,	32	10.5	32	10.5	1.9	8.1

Species	Strains	Description	MIC		MBC		SI	
			µg/mL	µM	µg/mL	µM	THP-1	HFF-1
		MEM R, PTZ R PB R, TET I, TIG R, SXT R						
	ACI40 [16]	Clinical strain. AK R, SAM R, FEP R, CTX R, CAZ R, CRO R, CIP R, CL S, CN R, IMI R, MEM R, PTZ R, PB S, TET R, TIG R, SXT R	32	10.5	32	10.5	1.9	8.1
	ACI42 [16]	Clinical strain. AK R, SAM R, FEP R, CTX R, CAZ R, CRO R, CIP R, CL S, CN R, IMI R, MEM R, PTZ R, PB S, TET R, TIG R, SXT R	32	10.5	64	21.1	1.9	8.1
	AM83*	Clinical strain, tracheal secretion isolate. AK R, SAM R, FEP R, CAZ R, CRO R, CL S	32	10.5	32	10.5	1.9	8.1
	AM87*	Clinical strain, urine isolate. AK S, SAM R, FEP R, CAZ R, CRO R, CIP R, CL S	32	10.5	32	10.5	1.9	8.1
P. aeruginosa	ATCC 27853	Clinical strain, blood isolate. Inducible AmpC	64	21.1	64	21.1	0.9	4
	RPPse09*	Clinical strain, rectal swab isolate. AK S, FEP S, CAZ S, CIP S, CL S, CN S, IMI R, MEM R, PTZ S	128	42.1	256	84.2	0.5	2
	RPPse07*	Clinical strain, urine isolate. AK R, FEP R, CAZ I, CIP R, CL S, CN R, IMI R, MEM R, PTZ I	64	21.1	256	84.2	0.9	4
	PSE6	*bla*$_{KPC}$	64	21.1	64	21.1	0.9	4
	PAO1		64	21.1	64	21.1	0.9	4

*, Ilana Camargo (LEMiMo); **, Ana Cristina Gales (LEMC/Alerta); American Type Culture Collection (ATCC). Sequence type (ST). R, resistant; S, sensitive, I, intermediate; Amikacin (AK), Amoxicillin-clavulanate (AMC), Ampicillin (AMP), Ampicillin-sulbactam (SAM); Aztreonam (ATM), Cefepime (FEP), Cefotaxime (CTX), Cefoxitin (FOX), Cefpodoxime (CPD), Ceftazidime (CAZ), Ceftriaxone (CRO), Cefuroxime (CXM), Ciprofloxacin (CIP), Chloramphenicol (CHL), Colistin (CL), Daptomycin (DAP), Erythromycin (ERY), Ertapenem (ETP), Gentamicin (CN), Imipenem (IMI), Linezolid (LNZ), Meropenem (MEM), Moxifloxacin (MXF), Nitrofurantoin (NIT), Piperacillin (PIP), Piperacillin-tazobactam (PTZ), Polymyxin B (PB), Tedizolide (TED), Teicoplanin (TEC), Tetracycline (TET), Tigecycline (TIG), Trimethoprim-sulfamethoxazole (SXT), Vancomycin (VAN)

Table S3. Synergism for p-BthW in *S. aureus* ATCC 25923 (MIC=16 mg/L).

Antibiotics	Antibiotics MIC (mg/L)	Combination (mg/L)		FIC Index
		MIC$_{ANTIBIOTIC}$	MIC$_{p\text{-}BthW}$	
Ciprofloxacin	0.5	0.5	16	2
Tobramycin	0.5	0.5	16	2
Daptomycin	1	1	16	2
Vancomycin	2	2	32	3
Ampicillin	0.06	0.06	16	2
Imipenem	0.015	0.015	16	2
